# Natural products for biocontrol of *Pseudomonas syringae*: mechanisms and applications

**DOI:** 10.3389/fpls.2026.1754522

**Published:** 2026-03-04

**Authors:** Xiaosen Han, Zihan Yang, Sai Jiang, Lei Meng, Lin Jiang

**Affiliations:** 1 School of Pharmacy, Hunan University of Chinese Medicine, Changsha, China; 2 Institute of Innovative Traditional Chinese Medications, Hunan Academy of Chinese Medicine, Changsha, China

**Keywords:** animal-derived compounds, biological control strategies, mechanisms of action, microbial-derived compounds, natural products, plant-derived compounds, Pseudomonas syringae

## Abstract

*Pseudomonas syringae* functions as a model phytopathogen causing numerous crop diseases, resulting in substantial economic losses in global agriculture. Presently, management of *P. syringae* predominantly depends on chemical pesticides; however, their prolonged application has contributed to escalating resistance and environmental contamination, highlighting urgent requirement for sustainable biological control approaches. In this review, we examine recent advances in the utilization and mechanistic understanding of natural products derived from plants, animals, and microorganisms for the control of *P. syringae.* Plant-derived compounds—including flavonoids, terpenoids, and alkaloids—inhibit *P. syringae* infection by targeting the bacterial type III secretion system (T3SS), disrupting cell membrane integrity, promoting reactive oxygen species (ROS) accumulation, and activating plant immune signaling pathways such as salicylic acid (SA) and jasmonic acid (JA) cascades. Animal-derived substances, such as chitosan, propolis, and antimicrobial peptides, primarily exert antibacterial effects through membrane disruption and immune system stimulation. Microbial-derived natural products contribute to synergistic disease suppression by modulating host immunity and interfering with the pathogen’s quorum sensing mechanisms. Evidence indicates that these natural products possess multi-target antimicrobial properties, offering a rich repository of candidate molecules, such as baicalein, lignans, and carvacrol, for the development of eco-friendly antibacterial agents. Future investigations should focus on detailed characterization of these bioactive compounds and their specific disease targets, optimization of extraction methodologies to improve stability and bioavailability, and comprehensive assessment of environmental safety to advance the industrial implementation of sustainable biocontrol strategies

## 1 Introduction

As a model plant pathogen, *Pseudomonas syringae* has been documented to infect over 180 species of economically significant crops, leading to diseases such as spot blotch, ulcers, and diamondback disease, which cause significant impact on the global agricultural economy annually (Shao et al., 2021). More than 60 pathogenic variants, or pathovars, of this bacterium have been characterized, each exhibiting specificity toward particular host plant species (Jin et al., 2003; Xin et al., 2018). These pathovars cause various plant diseases, including walnut leaf spot death (Keshtkar et al., 2016), bacterial wilt in Chinese mulberry (Choi et al., 2017), angular mottle disease in cucumber, and inflorescence rot in grapevines (Gerin et al., 2019). Its hosts include mulberry, apricot, plum, tomato, soybean, and Arabidopsis thaliana (Giovanardi et al., 2018). In China, infections caused by *P. syringae*, including bacterial ulcer in kiwifruit and bacterial perforation in cherries, occur with notable frequency, significantly constraining the advancement of specialized agricultural sectors (Vicente and Roberts, 2007; Chen et al., 2018). Furthermore, infection of fruits by *P. syringae* causes significant economic damage (George et al., 2021). Grapes account for nearly a quarter of global fruit production. In a study by D. Gerin et al., bunch rot caused by *P. syringae* led to the loss of grape inflorescences, ultimately resulting in a reduction of fruit set by up to 60% of the original yield (Gerin et al., 2019). Sweet cherry is a high-value global fruit worldwide. In the 2023–2024 season, Chile exported 413, 979 tons. However, bacterial canker caused by *P. syringae* resulted in yield losses of 10% to 40% in commercial orchards and nurseries, resulting a severe financial losses for the cherry industry (Carreras et al., 2024). Pepper is a major crop globally. In 2020, the United States produced 4.7 million pounds of peppers, valued at $579 million. Yet, *P. syringae* causes severe dark necrotic lesions on pepper leaves, significantly reducing crop yield and quality, which would lead to considerable economic losses (Ranjit et al., 2024).

Currently, chemical agents remain the main strategy for the prevention and management of Pseudomonas butyrica. However, their long-term and widespread has resulted in frequent mutations within resistance genes of the pathogen, including those encoding efflux pumps such as *mexB* (Helmann et al., 2019). Additionally, the accumulation of pesticide residues in the food chain poses a serious ecological threat (Bu et al., 2014). Bu Yuanqing et al (Bu et al., 2014). reported that organophosphorus pesticide residues in soils from major agricultural regions in China ranged between 0.03 and 0.52 mg/kg, substantially surpassing the safety limits established by the European Union. To overcome these problems, the development of environmentally sustainable control strategies using natural products has become a major research focus worldwide (Alexandra et al., 2016; Hardian and Wiwiek, 2016). Specifically, current major chemical or non-natural control strategies have serious drawbacks. For example, Fosetyl-aluminum is a synthetically produced organophosphorus bactericide that exhibits significant inhibitory effects against *P. syringae*. However, its use can increase the diversity of existing plant pathogens, thereby causing complications in pathogen identification, loss of resistance in plant varieties, and increased damage from diseases they induce (Butsenko et al., 2020). In contrast, metal and metal oxide nanoparticles demonstrate potent bactericidal activity through multiple mechanisms, offering high efficacy and persistence. However, their production is challenging. The sourcing of biological materials and subsequent purification in bioreactors are complex and expensive processes. Additionally, they have drawbacks like the potential toxicity of the chemicals used and the lower biocompatibility of the resulting products (You et al., 2024). Moreover, the continuous use of streptomycin bactericides exerts directional selection pressure on pathogen populations, leading to alterations in a significant proportion of plasmid or chromosomal genotypes, which consequently promotes the gradual development of drug resistance in *P. syringae* (Sundin et al., 1994). Although the deployment of natural products does not necessarily preclude the emergence of resistance, they are less prone to inducing resistance compared to traditional bactericides.

Natural products show great potential for controlling plant diseases owing to their ability to target multiple pathways and their environmental compatibility. For instance, flavonoids can protect plants by inhibiting the type III secretion system (T3SS) of pathogens (He et al., 2023)and activating the phytosalicylic acid (SA) signaling pathway (Li et al., 2019). Similarly, secondary metabolites produced by microorganisms have been shown to regulate both the host immune response and the pathogen community sensing mechanisms (Zhang et al., 2019). In a collaborative study between Lei’s and Zhou’s research teams, it was identified that erucic acid amide, a natural compound from Arabidopsis thaliana, specifically targets HrcC, a critical component of the pathogen’s T3SS. HrcC functions to block the translocation of effector proteins into host cells, and the application of erucic acid amide achieved a preventive efficacy of 72.3% in field trials (Miao et al., 2025). This finding offers a direct molecular target for the development of novel bacterial inhibitors.The FDA clearly stipulates that if a natural compound is to be developed as a drug, it must comply with the “Guidance for Industry: Botanical Drug Development.” Companies can submit an Investigational New Drug (IND) application to conduct Phase I and II clinical trials. After accumulating data, they can proceed to later-stage clinical studies, thoroughly validating safety at each stage of the trials, investigating adverse reactions at different dosages, and ensuring that benefits outweigh risks before obtaining market approval via a New Drug Application (NDA). Alternatively, market entry can be achieved through the Over-the-Counter (OTC) Monograph pathway. This requires publicly available data (including results from adequate and well-controlled clinical studies) demonstrating safety and effectiveness, and an application must be submitted to amend the monograph to include the natural compound ingredient. Examples such as psyllium and senna have been incorporated into the OTC review category.

For clarity, we adopt the following three-tiered classification based on international regulatory frameworks: (1) Natural pesticides refer to substances derived directly from living organisms or minerals without chemical modification, as recognized in EU Regulation 1107/2009 and by the U.S. EPA’s Biopesticide division; (2) Semi-synthetic pesticides are naturally derived compounds chemically altered to enhance stability or efficacy, regulated under conventional frameworks such as those in Australia and Canada; and (3) Synthetic pesticides are fully chemically synthesized compounds subject to stringent global risk assessments by bodies like EFSA and WHO. This classification underscores that “natural” and “chemical” are not mutually exclusive but exist along a spectrum defined by the origin and processing of active ingredients (Copping and Menn, 2000). For example, while natural pesticides are obtained via physical extraction, semi-synthetic ones like pyrethroids involve targeted chemical modifications of natural precursors (Khambay and Jewess, 2005). Crucially, the legal recognition and regulatory pathways for these categories vary across jurisdictions: the U.S. EPA provides streamlined reviews for biopesticides, the EU’s Regulation 1107/2009 sets specific “low-risk” criteria for natural-origin substances, and major economies such as China and Brazil have implemented prioritized registration systems for biological products (European Parliament and Council, 2009; Ministry of Agriculture and Rural Affairs of the People’s Republic of China, 2021; Brazilian Ministry of Agriculture, Livestock and Supply, 2022; U.S. Environmental Protection Agency, 2023) Thus, regulatory acceptance depends not merely on a product’s “natural” label but on a holistic assessment of its origin, degree of modification, and overall risk profile.

This review systematically examined the bacteriostatic mechanisms of natural products sourced from plants, animals, and microorganisms. The interactions between flavonoids, terpenoids, and other bioactive compounds with pathogenic bacteria were analyzed, with the objective of offering theoretical insights and establishing a candidate compound repository to facilitate the development of sustainable strategies for agricultural disease prevention and control.

## 2 Prevention and management of *Pseudomonas syringae* through natural plant-derived compound

### 2.1 Flavonoids

#### 2.1.1 Flavonoids

Baicalein, a 5, 6, 7-trihydroxyflavone extracted from the roots of *Scutellaria baicalensis* (Labiatae family), has been shown to exhibit significant antioxidant and antibacterial properties. Studies show that baicalein triggers an early immune response by activating plant NADPH oxidase (RBOH)-mediated production of reactive oxygen species (ROS). In the *Arabidopsis*-*Pseudomonas syringae* interaction model, application of baicalein at a concentration of 100 μmol/L resulted in a 2.1-fold increase in leaf hydrogen peroxide (H_2_O_2_) levels within six hours, which was accompanied by a rapid upregulation of defense-related genes *FRK1* and *WRKY22* (Wang et al., 2022). Furthermore, baicalein was found to inhibit extracellular polysaccharide (EPS) synthesis by 42%, achieved through the suppression of *lasI* gene expression, a critical component of the quorum sensing system in pathogenic bacteria, thus impairing their capacity for biofilm formation (Zhang et al., 2021).

Luteolin (5, 7, 3’, 4’-tetrahydroxyflavone) a flavonoid abundant in Asteraceae and Umbelliferae plants. Its antimicrobial activity is primarily attributed to the targeted inhibition of the type III secretion system (T3SS) in Pseudomonas aeruginosa, as shown by He et al (He et al., 2023). through the application of a dual luciferase reporter assay. Specifically, it suppresses the promoter activities of *P. syringae* genes *hrpL* and *hrpW* by 63% and 58%, respectively. The *hrpL* gene codes for the master regulator of the T3SS gene cluster. Further study revealed that luteolin impedes the interaction with the *hrpL* promoter by binding to the N-terminal structural domain of the HrpS protein, exhibiting a dissociation constant (KD) of 32.6 μmol/L. This binding event culminates in a 71% decrease in the secretion of the effector protein AvrPto (He et al., 2023). In the context of plant immune responses, luteolin enhances salicylic acid (SA) levels in Arabidopsis leaves by approximately 1.8-fold through the upregulation of SA biosynthetic genes *ICS1* and *PAL1*. Furthermore, it induces a sustained elevation in the expression of systemic acquired resistance (SAR) marker genes *PR-1* and *PR-5* (Li et al., 2019).

Apigenin, a structural analogue of luteolin distinguished by the absence of a hydroxyl group at the C3′ position, acts its antibacterial effects primarily through the enhancement of plant cell wall integrity. Zhang et al (Zhang et al., 2018). demonstrated that pre-treatment with 50 μmol/L apigenin resulted in a 35% increase in lignin content within Arabidopsis leaves and increased callose deposition 2.3-fold over control levels. This effect was mediated by the activation of phenylalanine ammonia-lyase (PAL) and peroxidase (POD) enzymatic activities. Transcriptomic analysis further indicated that apigenin treatment upregulated the expression of *PR-1* and *PR-2* genes by 3.5-fold and 2.8-fold, respectively. Importantly, this induction was entirely lost in the salicylic acid (SA) signaling pathway mutant *NahG*, confirming that apigenin′s effect requires an intact the SA-mediated defense pathway (Zhang et al., 2020).

#### 2.1.2 Flavonols

Quercetin (3, 3’, 4’, 5, 7-pentahydroxyflavone), a ubiquitous plant flavonoid, strongly inhibits *P. syringae* (MIC = 0.0121 μmol/mL) (Qin et al., 2009). Qin et al (Qin et al., 2009). reported that quercetin disrupts the pathogenic bacterial cell membrane, leading to a 3.2-fold increase in nucleic acid leakage measured at 260 nm within a 2-hour period. In terms of plant defense mechanisms, pretreatment with quercetin elicited a hydrogen peroxide (H_2_O_2_)-dependent hypersensitive response (HR) in Arabidopsis thaliana and upregulated the expression of critical genes involved in the salicylic acid (SA) signaling pathway, specifically *PAD4* and *NPR1* (Jia et al., 2010). Importantly, quercetin-induced resistance was maintained in *jarl* (jasmonic acid signaling mutant) and *ein2* (ethylene signaling mutant) plants, whereas it was completely abolished in *NahG* and *npr1* mutants, indicating that this resistance is specifically mediated through the SA-NPR1 signaling pathway (Jia et al., 2010).

Kaempferol (3, 5, 7, 4’-tetrahydroxyflavone) is frequently present in plant extracts alongside quercetin, with both compounds exhibiting synergistic effects that enhance antimicrobial activity via multiple target pathways. Zareath et al (Teran Baptista et al., 2020). successfully isolated a quercetin-kaempferol complex from an ethyl acetate extract of Brazilian pepper (Schinus terebinthifolia), which demonstrated a markedly lower minimum inhibitory concentration (MIC) against *P. syringae* (0.0121 mg/mL) relative to the MIC values of the individual flavonoids (0.028 mg/mL for quercetin and 0.035 mg/mL for kaempferol) (Gomes et al., 2020). Further mechanistic studies suggest that kaempferol contributes to the stabilization of immunoreceptor proteins by suppressing the transcription of the type III secretion system (T3SS) effector protein hopQ1 in the pathogenic bacterium, thereby diminishing its interaction with the plant’s E3 ubiquitin ligase (Teran Baptista et al., 2020).

Myricetin (3, 5, 7, 3’, 4’, 5’-hexahydroxyflavone) exhibits strong antioxidant activity, attributed to its multiple B-ring hydroxyl groups. According to Tingting Liu and colleagues (Zhang et al., 2008), the effects of myricetin can be inhibited by introducing a PIP-box cis-element into the promoter region of the hpL gene in *P. syringae*. The binding efficiency of the *HrpL* factor results a 56%-72% reduction in expression of the T3SS structural genes *hrcC* and *hrpA.* In addition, myricetin activates the plant MAPK signaling pathway, leading to a 2.4-fold increase in the phosphorylation of MPK3/6, and stimulates the increased expression of reactive oxygen species (ROS) scavenging enzymes like superoxide dismutase (SOD) and catalase (CAT), which alleviates oxidative damage caused by pathogen infection (Zhang et al., 2008).

#### 2.1.3 Isoflavonoids

Genistein (5, 7, 4′-trihydroxyisoflavone), is an isoflavonoid compound predominantly found in leguminous plants. It exerts antibacterial activity by interfering with the bacteria two-component signal system (TCS). Wang Zhuoya et al (Wang et al., 2022). used a β-galactosidase reporter assay to show that genistein at a concentration of 150 μmol/L specifically inhibits the expression of the small RNA rsmZ, which is regulated by the gacS/gacA system, inhibiting rsmZ expression by 63%. As a result, transcription of the coronatine synthesis gene *cfa* was reduced by 48%. In terms of plant immune response activation, genistein induces isoflavone synthase (IFS) activity in soybean leaves, leading to glyceollin accumulation of 127 μg/g fresh weight within 48 hours. This elevation significantly suppresses the colonization of *P. syringae* pv. *syringae*, as evidenced by a 91% reduction in bacterial colony counts (Liu et al., 2016).

Daidzein (7, 4’-dihydroxyisoflavone), a metabolic precursor of genistein, primarily enhances plant cell wall defenses. *In vitro* experiments demonstrated that exposure to 200 μmol/L daidzein decreased the membrane potential of *P. syringae* by 35 mV and induced a 2.8-fold increase in ATP leakage (Liu and Chen, 2015). Furthermore, in transgenic soybean plants, daidzein treatment led to a 5-fold, 3-fold, and 4-fold upregulation in the expression levels of *PR-1*, *PR-2*, and *PR-5* genes, respectively, and resulted in a 60% reduction in lesion size relative to untreated controls (Graham and Graham, 1996).

#### 2.1.4 Flavanols

Catechin, a flavan-3-ol polyphenol characterized by the chemical structure (2R, 3S)-3, 3, 4, 5, 7-pentahydroxyflavan, is abundantly present in tea, cocoa, and red wine. This compound exhibits antibacterial activity against *P. syringae* via two primary mechanisms: direct antimicrobial effects and activation of the host immune response. *In vitro* studies demonstrate that catechins inhibit topoisomerase activity by intercalating into the pathogen’s DNA double helix, with a binding constant (Kb) of 1.2 × 10^4 L·mol^−1, thereby obstructing DNA replication (Wang and Wu, 2019). In Arabidopsis thaliana, exposure to 0.1 mmol/L catechin resulted in a 2.5-fold increase in reactive oxygen species (ROS) fluorescence within leaf blades, a response contingent upon the oxidative burst mediated by plasma membrane-localized RBOHD proteins (Zhang et al., 2021). Furthermore, transcriptomic analysis conducted by Wang et al (Wang and Wu, 2019). revealed the upregulation of critical genes associated with phenylpropanoid metabolism, including *PAL1* and *C4H*, following catechin treatment. Correspondingly, the concentration of the phytoalexin camalexin was elevated by 2.8-fold, indicating that catechin enhances plant disease resistance through modulation of secondary metabolic pathways.

**Figure 1:**
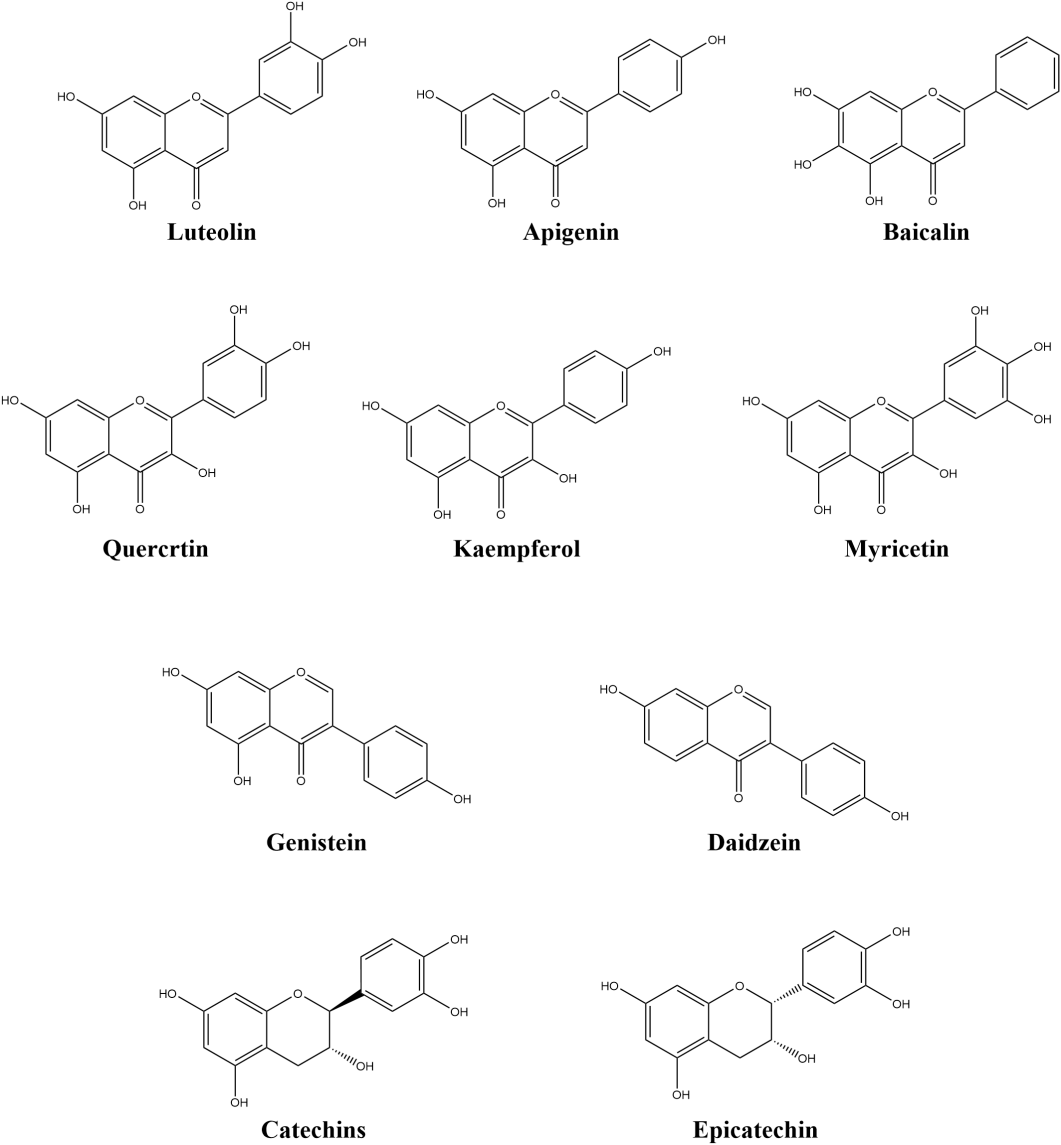
Chemical structure of flavonoids.

Epicatechin (**Figure 1**), a member of the flavan-3-ol subgroup, has an MIC of 0.15 mg/mL against *P. syringae*. It kills bacteria by inhibiting DNA rotamase (IC_50_ = 28 μmol/L) and topoisomerase IV (Zhang and Wang, 2014). In tomato plants, epicatechin enhances systemic acquired resistance (SAR) by upregulating the expression of *LOX2* and *AOS*, which are critical genes in the jasmonic acid (JA) signaling pathway. Additionally, it elevates lipoxygenase activity by 1.7-fold, which strengthen plants defense (Dong et al., 2021).

### 2.2 Terpenoids and volatile oils

#### 2.2.1 Terpenoids

##### 2.2.1.1 Terpenoids

Terpenoids are a large class of plant-derived natural compounds with a wide range of biological activities. They are classified based on the number of isoprene units as monoterpenes, sesquiterpenes, diterpenes, etc. They combat bacteria by directly damaging pathogen structures (e.g., cell membranes) and by tuning plant immune signaling. These properties underscore their considerable potential in the prevention and management of *P. syringae* infections.

Limonene, a 1, 8-terpene diene chemical structure, is a major component of citrus peel essential oils. It has significant antibacterial and anti-inflammatory activities. The antibacterial effect of limonene stems mainly from its ability to disrupt bacterial cell membrane integrity. For instance, limonene contained within the essential oil of *P. syringae* markedly enhances the permeability of the bacterial cell membrane. Following a 90-minute exposure to 2000 μg/mL of limonene, the electrical conductivity (EC) of the pathogen increased by a factor of 3.2 relative to the control, indicating extensive leakage of intracellular material (Elshafie et al., 2019). Furthermore, limonene has been shown to attenuate the expression of virulence factors by inhibiting the pathogen’s quorum sensing system (Elshafie et al., 2019).

Carvacrol (2-methyl-5-isopropylphenol) is the primary active ingredient in Labiatae family essential oils, accounting for 35–42% of the oil in species such as winter savory, mint, and calyx mint (Ghahari et al., 2017). Carvacrol combats *P. syringae* via two primary mechanisms: First, it directly inhibits the pathogen. By integrating into the bacterial membrane via hydrophobic interactions, it reduces membrane potential and causes ATP leakage. Its MIC against *P. syringae* is 0.08 mg/mL (Oliveira-Fernandes et al., 2023). Second, it modulates plant immunity by suppressing pathogen-induced ABA accumulation and alleviating ABA-mediated repression of JA signaling, which restores plant defense (Oliveira-Fernandes et al., 2023).

##### 2.2.1.2 Sesquiterpenes

β-Caryophyllene, a bicyclic sesquiterpene, is widely in plants including lemon and nutmeg. Studies show that β-caryophyllene boasts disease resistance by modulating plant secondary metabolism. For instance, the overexpression of the *LaMYC7* gene in tobacco plants led to a marked increase in stigmasterol biosynthesis and a 62% decrease in *P. syringae* colonization (Dong et al., 2024). Furthermore, in Arabidopsis thaliana, the *AtMYC2* transcription factor promotes stigmasterol production by binding to the promoter region of *TPS21/11*, which triggers a reactive oxygen species (ROS) burst (Hong et al., 2012).

**Figure 2:**
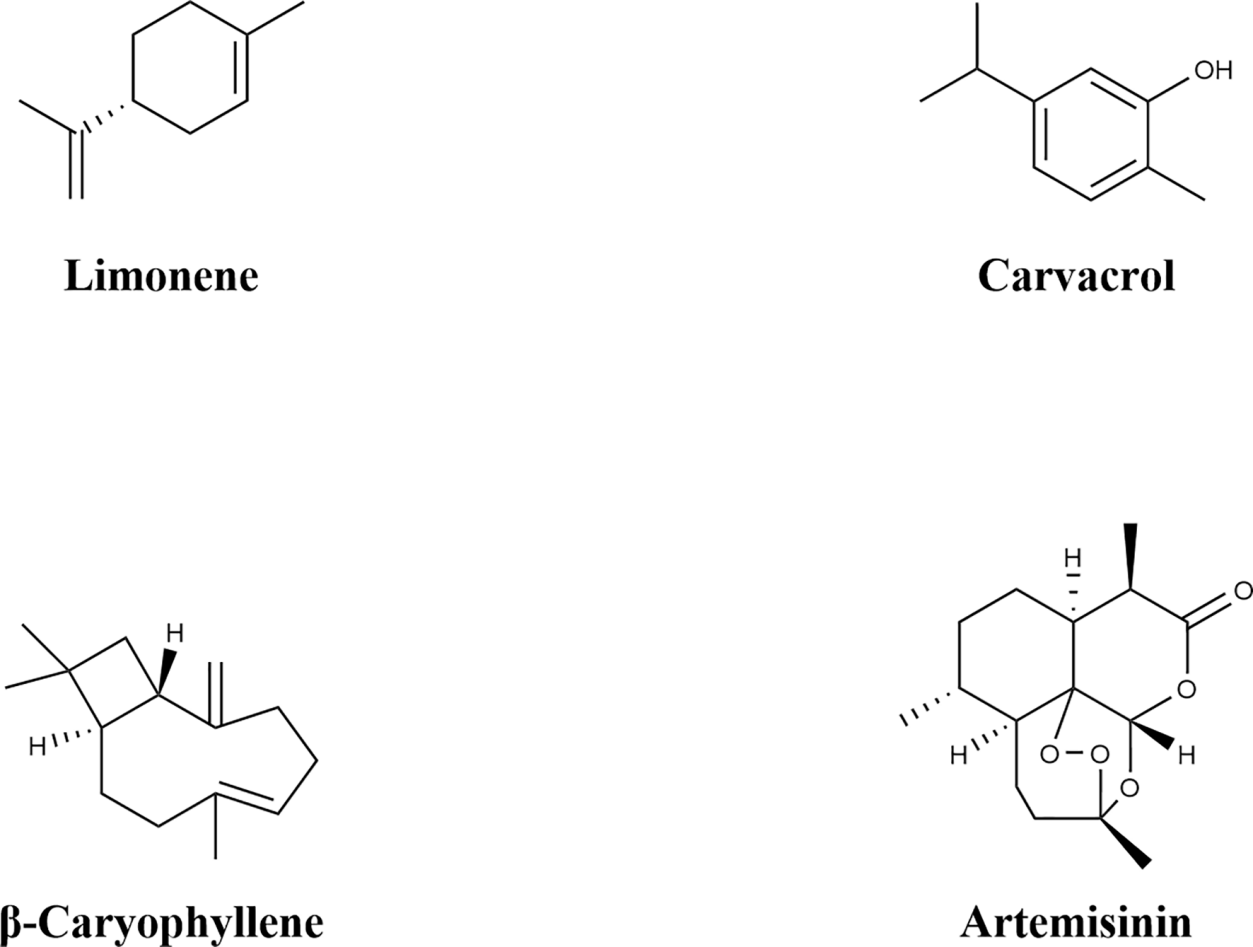
Chemical structure of terpenoids.

Artemisinin (**Figure 2**), a sesquiterpene lactone (C_15_H_22_O_5_), was first isolated from Artemisia annua. Its resistance mechanism involves activating host immune responses. Specifically, the transcription factor *AaWRKY17* is inducible by artemisinin and subsequently enhances the expression of PR5 (pathogenesis-related protein 5) and NHL10 (NDR1/HIN1-like protein 10). This enhances Arabidopsis resistance to *P. syringae* by 4.1-fold (Chen et al., 2021).

#### 2.2.2 Volatile oils

Thyme oil is an essential oil with strong antiseptic, antibacterial, and antiviral activities. It has demonstrated efficacy in addressing conditions such as dandruff and hair loss and is frequently utilized in the treatment of respiratory ailments including colds, coughs, and sore throats. The essential oil of thyme is rich in terpenes. Within common thyme, six principal chemotypes have been identified, its major chemotypes contain phenolic compounds like thymol and carvacrol, as well as non-phenolics like linalool, which confer its antibacterial and fungicidal properties. These constituents contribute to the antibacterial and fungicidal properties of thyme essential oil. Specifically, the minimum inhibitory concentration (MIC) of thyme essential oil against *P. syringae* is 0.125 μL/mL (Kosakowska et al., 2024).

Cinnamon oil is a volatile essential oil obtained through steam distillation of the dried branches and leaves of Cinnamomum camphora. This oil appears as a clear yellow to yellow-brown liquid, characterized by a distinctive aroma and a sweet, spicy flavor profile. In traditional Chinese medicine and culinary practices, it is used for its warming and stomachic properties. The essential oil is notably rich in cinnamaldehyde, a compound active against the bacterium *P. syringae* (Jung et al., 2024). It works by modifying specific amino acid side chains within bacterial proteins, which inhibits bacterial growth. Furthermore, the incorporation of various substituent groups into benzene rings, heterocyclic structures, and aliphatic chains results in differential inhibitory effects. Notably, the presence of monosubstituted electron-donating groups has been shown to markedly increase the bacteriostatic potency of these compounds (Liu et al., 2024).

### 2.3 Alkaloids

Alkaloids are nitrogen-containing heterocyclic compounds found widely in plants, animals, and microbes, with diverse biological activities. In investigations involving *P. syringae*, alkaloids can disrupt bacterial virulence systems, compromise cell integrity, and modulate plant immune responses. Their structural diversity and varied mechanisms make alkaloids promising leads for developing new antibacterial agents.

#### 2.3.1 Isoquinoline alkaloids

Berberine (BBR) is a typical isoquinoline alkaloid with the structure 5, 6-dihydro-9, 10-dimethoxybenzo[g]-1, 3-benzodioxolane[5, 6-a]quinolizine. It is mainly extracted from plants like Coptis chinensis and Phellodendron chinense. The antimicrobial activity of berberine against *P. syringae* mainly results from its ability to inhibit efflux pump function. Tyler C. Helmann et al (Helmann et al., 2019)created a transposon mutant library of *P. syringae* B728a, which reveal that deletion of the endosomal membrane transporter protein Psyr_0541 markedly enhanced bacterial susceptibility to berberine, as shown by a 42% reduction in the minimum inhibitory concentration (MIC). This finding confirms that Psyr_0541 modulates bacterial sensitivity to berberine via efflux. Furthermore, berberine has been shown to activate plant immune responses by promoting the accumulation of reactive oxygen species (ROS). For instance, in Arabidopsis thaliana, berberine treatment induced a 2.1-fold increase in RBOH gene expression and a 1.8-fold elevation in hydrogen peroxide (H_2_O_2_) levels 24 hours following application (Zhang et al., 2021).

#### 2.3.2 Pyrrolizidine alkaloids

Nicotine, a pyrrolizidine alkaloid from the Solanaceae family, has the structure (S)-3-(1-methylpyrrolidin-2-yl) pyridine. Its antibacterial properties extend beyond direct antimicrobial activity to include the enhancement of disease resistance through modulation of phytohormone signaling pathways. Lu Zhang et al (Zhang et al., 2012). demonstrated that silencing the alternative oxidase (AOX) gene in transgenic tobacco plants led to an accelerated cell death phenotype upon pathogen infection in irAOX lines. Furthermore, the concentrations of salicylic acid (SA) and hydrogen peroxide (H_2_O_2_) in the leaves increased by factors of 2.3 and 3.1, respectively, relative to wild-type plants. These findings suggest that nicotine may confer disease control by inducing systemic acquired resistance (SAR) via an SA-dependent signaling mechanism. *In vitro* assays revealed that the minimum inhibitory concentration (MIC) of nicotine against *P. syringae* was 0.8 mmol·L^-^¹, and its effectiveness increased at higher pH (Zhang et al., 2012).

#### 2.3.3 Other alkaloids

**Figure 3:**
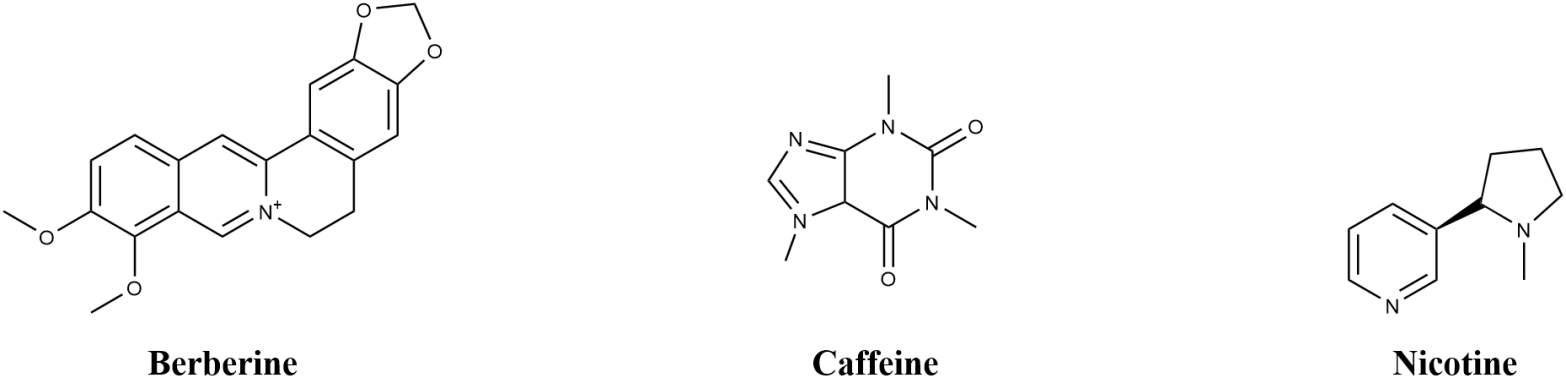
Chemical structure of alkaloid compounds.

Caffeine (**Figure 3**), a purine alkaloid with the structure 1, 3, 7-trimethylxanthine, is commonly found in coffee (Coffea arabica), tea (Camellia sinensis), and various other plant species. Wojciech Sledz and colleagues (Sledz et al., 2015) investigated the antimicrobial activity of caffeine against *P. syringae using* the micro-broth dilution method. Their investigation established the minimum inhibitory concentration (MIC) and minimum bactericidal concentration (MBC) of caffeine against *P. syringae* as 5.0 ± 0.1 mg·mL^-^¹ and 43.3 ± 5.77 mg·mL^-^¹, respectively, indicating a bactericidal effect that is dependent on concentration. Mechanistic studies show that caffeine disrupts bacterial function through three main pathways (1): inhibition of RNA polymerase activity, leading to a 62% reduction in *hrpL* gene transcription (Zhao and Zhou, 2021) (2).; enhancement of cell membrane permeability, indicated by a 3.5-fold increase in nucleic acid leakage (measured at A260); (3) interaction with FtsZ proteins, which are critical for bacterial cytokinesis, thereby delaying cell division (Zhao and Zhou, 2021).

### 2.4 Phenolic compounds

#### 2.4.1 Phenolic compounds

P-coumaric acid (4-hydroxycinnamic acid) is a phenolic acid commonly present in various plants, including wheat, corn, and soybeans. Its structure consists of a benzene ring with a hydroxyl group at C4 and an acrylic acid side chain. It plays a dual role as a plant cell wall component and a key signaling molecule in defense. Wang Xiaojie et al (Wang, 2017). demonstrated that p-coumaric acid exerts a concentration-dependent bidirectional regulatory effect on *P. syringae*. Specifically, at low concentrations (<0.5 mmol·L^-^¹), it promotes bacterial growth through mechanisms that remain unclear, whereas at higher concentrations (≥1.0 mmol·L^-^¹), it inhibits bacterial activity. The most sensitive strains were biovar4 and biovar2, with MICs of 0.8 mM and 1.0 mM, respectively. During plant-pathogen interaction, free phenolic acid levels initially decline, partially due to pathogen consumption. The remaining p-coumaric acid is then channeled by phenylalanine ammonia-lyase (PAL) into lignin biosynthesis, reinforcing cell wall integrity and forming a physical barrier (Wang, 2017).

#### 2.4.2 Tannins

Tannins are a group of complex polymers rich in phenolic hydroxyl groups, widely distributed in plants such as coffee, tea, and walnuts. Chemically, they are classified into hydrolyzable and condensed tannins. Canzoniere et al (Canzoniere et al., 2021). reported that tannins exert dual antibacterial effects on Pseudomonas fragans. They first interact with membrane proteins via hydrophobic interactions, impairing membrane integrity and inhibiting bacterial adhesion. Secondly, tannins selectively downregulate the expression of hrpL, a key gene in the Type III Secretion System (T3SS), reducing its transcription by 42–67%. Additionally, they competitively inhibit the binding of the quorum-sensing molecule N-acyl homoserine lactone (AHL) to its receptor, with a competitive inhibition constant (Ki) of 23.5 μmol·L^-^¹. Together, these mechanisms effectively disrupt the pathogen’s virulence regulatory network (Canzoniere et al., 2021).

Tannic acid, a typical hydrolyzable tannin, contains multiple adjacent phenolic hydroxyl groups in its molecular structure that act as signaling molecules to modulate bacterial pathogenicity. Using a fluorescent reporter gene system, Xie et al (Xie et al., 2021). showed that exposure to 2.5 μmol·L^-^¹ tannic acid upregulated the expression of the *P. syringae* rhpR-lux fusion gene by sixfold and enhanced autophosphorylation of the histidine kinase RhpS, increasing the logarithmic growth rate by 2.3-fold. Subsequent mechanistic studies revealed that tannins bind to a conserved cysteine residue (Cys201) in the sensing domain of RhpS, disrupting its phosphatase activity. This interaction sustains phosphorylation of the response regulator RhpR, ultimately suppressing transcriptional activation of T3SS genes (Xie et al., 2021).

#### 2.4.3 Resveratrol

**Figure 4:**
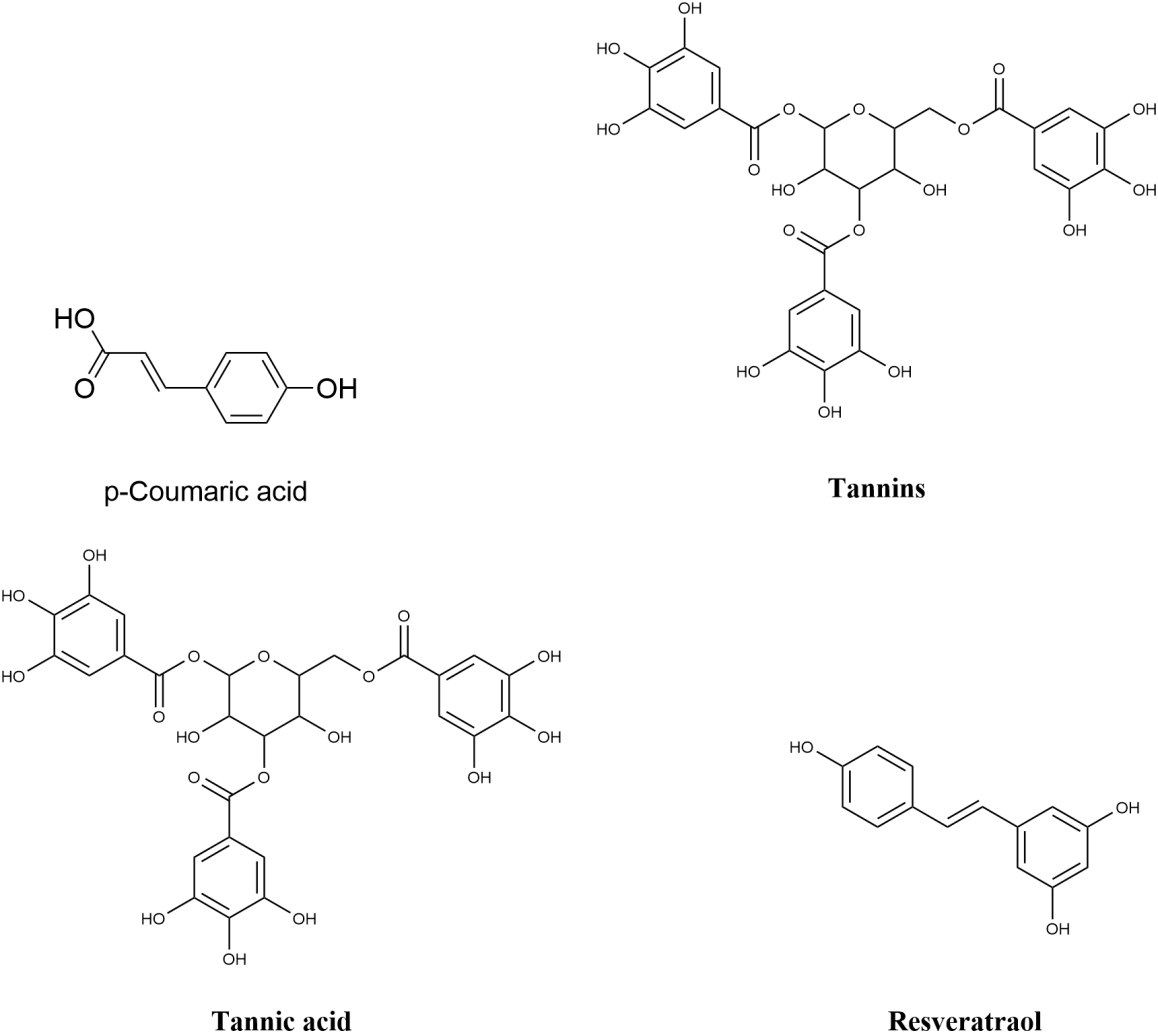
Chemical structure of other class compounds of plant origin.

Resveratrol (**Figure 4**) is a polyphenolic compound primarily found in grape skins and peanut seed coats, with trans-isomers exhibiting superior biological activity. Kang et al (Kang et al., 2020). isolated resveratrol oligomers—specifically dimers (ε-viniferin) and trimers (miyabenol C)—from grapevine extracts using ultra-high-performance liquid chromatography-mass spectrometry (UHPLC-MS). Their results showed that these oligomers exerted significantly stronger inhibitory effects on the Type III Secretion System (T3SS) of Pseudomonas butyrica compared to monomers. The half-maximal inhibitory concentrations (IC_50_) were 12.3 μmol·L^-^¹ for dimers, 8.7 μmol·L^-^¹ for trimers, and 35.6 μmol·L^-^¹ for monomers.Molecular docking analyses revealed that these oligomers formed hydrogen bonds with the σ54-binding domain of the HrpL protein, specifically at residues Arg247 and Asp289. This interaction stabilized the domain and blocked its binding to the hrp regulator promoter region, thereby reducing the secretion of effector proteins (AvrPto and HrpZ) by 58–73% (Kang et al., 2020). Additionally, resveratrol oligomers synergistically enhanced systemic acquired resistance (SAR) in plants by activating the NADPH oxidases RBOHD and RBOHF. This activation triggered a reactive oxygen species (ROS) burst, characterized by a 2.1-fold increase in hydrogen peroxide (HE_2_O_2_) levels, and an 1.8-fold upregulation of salicylic acid (SA) accumulation (Kang et al., 2020).

### 2.5 Polysaccharides

**Figure 5:**
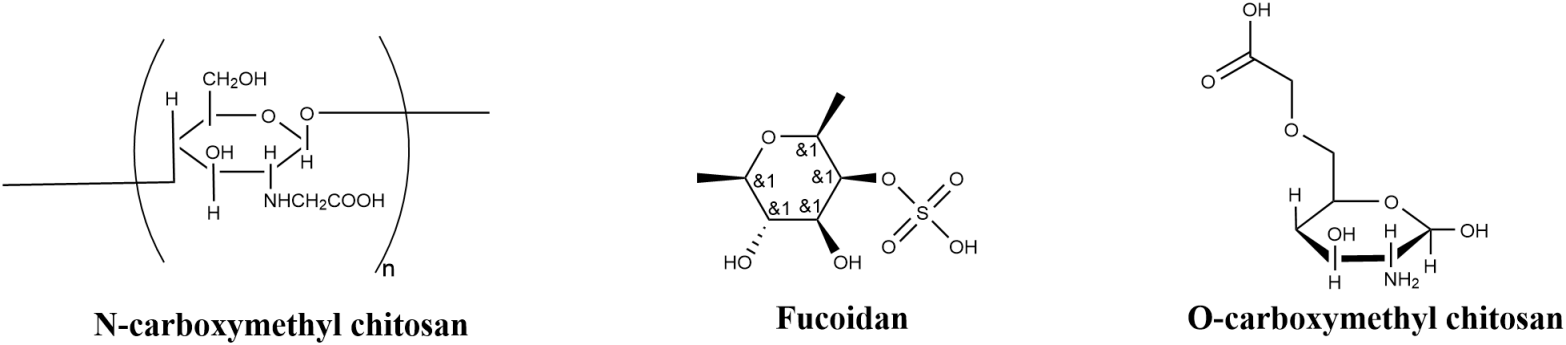
Chemical structure of polysaccharide compounds.

Fucoidan (**Figure 5**), is a sulfated polysaccharide derived from brown algae, composed of α-L-fucose linked via 1, 3- or 1, 4-glycosidic bonds and containing 17–34% sulfate groups. Studies have demonstrated that fucoidan exerts dual defense effects against plant pathogens. It directly interferes with bacterial quorum sensing by competitively inhibiting the binding of N-acylhomoserine lactone (AHL) signaling molecules to their receptors, thereby reducing the expression of virulence factors (e.g., extracellular polysaccharides, proteases) in *P. syringae* by 62–78% (Wang et al., 2021). Additionally, fucoidan activates the plant mitogen-activated protein kinase (MAPK) signaling pathway and induces synergistic responses of salicylic acid (SA)- and jasmonic acid (JA)-mediated defense pathways to suppress pathogens.For instance, transcriptomic analysis by Wang et al (Wang et al., 2023). showed that treating Arabidopsis thaliana with 0.2 mg/mL fucoidan upregulated the expression of WRKY33 and MYC2 transcription factors by 5.2- and 3.8-fold, respectively, while decreasing leaf pathogen colonization density by 89%. Notably, the antibacterial activity of fucoidan is structure-dependent: its biological efficacy declines significantly when the sulfate group content drops below 15% (Ale and Meyer, 2022).

### 2.6 Other classes of compounds

Juglone is an orange-yellow needle-like crystalline compound extracted from plants such as Juglans mandshurica. Chemically defined as 5-hydroxy-1, 4-naphthoquinone, it possesses antibacterial, anti-inflammatory, and anti-tumor activities. Studies have shown that juglone inhibits *P. syringae* infection through multiple pathways. First, it impairs the pathogen’s cell membrane integrity: via membrane potential (MP) detection, Han et al. found that juglone significantly reduced *P. syringae*’s MP, leading to leakage of intracellular proteins and nucleic acids and subsequent disruption of cell structure (Han, 2022). Second, it induces reactive oxygen species (ROS) accumulation, elevating intracellular ROS levels in the pathogen to trigger lipid peroxidation and protein oxidative damage, ultimately resulting in cell apoptosis (Liu, 2018). Third, it interferes with gene expression by intercalating into DNA molecules, disrupting nucleic acid metabolism. Notably, other studies have highlighted that juglone’s inhibitory effect on *P. syringae* is concentration-dependent—higher concentrations correlate with more pronounced degradation of the pathogen’s nucleic acids (Zhang, 2023).

Glucosinolates, sulfur-containing glycosides abundant in Brassicaceae plants, exhibit diverse biological activities in planta. Their degradation products (e.g., isothiocyanates, indole compounds) possess significant antibacterial properties, along with antioxidant, anti-inflammatory, and anti-cancer effects. They act through two primary mechanisms: direct inhibition of pathogen growth and regulation of plant disease resistance signaling pathways. When plants sustain damage, glucosinolates are hydrolyzed by myrosinase into active substances that disrupt the cell membrane structure of *P. syringae*. Additionally, Zhang et al (Zhang, 2023). demonstrated that nitrilases (NIT1/2/3) in Arabidopsis thaliana can catalyze the conversion of aliphatic glucosinolate degradation products into carboxylic acids. These carboxylic acids then activate salicylic acid (SA)-mediated systemic acquired resistance (SAR), significantly enhancing plant resistance to *P. syringae* pv. tomato (Pst) DC3000.

Anthocyanins (also known as anthocyans) are water-soluble natural pigments widely distributed in plants, consisting of colored aglycones derived from anthocyanidin hydrolysis. Their antibacterial mechanisms mainly involve three aspects: first, they bind to bacterial lipopolysaccharides via hydrophobic interactions, inhibiting cell wall formation and disrupting cell wall synthesis (Fang et al., 2015); second, the phenolic hydroxyl groups in their molecular structure efficiently scavenge ROS, alleviating plant oxidative stress—for example, purple onion anthocyanins and cyanidin 3-O-glucoside exhibited concentration-dependent free radical scavenging activity in experiments, with a maximum scavenging rate exceeding 85% (Li, 2019); third, anthocyanin treatment reduces the excessive accumulation of reductive substances in plants infected by Pst DC3000, thereby maintaining redox homeostasis and enhancing plant immune capacity (Chen, 2014).

Erucylamide, chemically named cis-13-docosenamide, is biocompatible and biodegradable, with multiple biological activities including antibacterial, anti-inflammatory, lipid-regulating, anti-tumor, and neuroprotective effects. Its antibacterial mechanism against *P. syringae* involves interference with the bacterial Type III Secretion System (T3SS) (Miao et al., 2025). As a core system enabling most animal and plant pathogenic bacteria to secrete toxic effector proteins into host cells, T3SS is critical for bacterial pathogenicity. Studies confirm that erucylamide specifically binds to HrcC, a key T3SS component, interfering with the protein’s correct localization on the pathogen’s outer membrane. This disruption effectively inhibits T3SS assembly, ultimately preventing pathogenic infection.

**Figure 6:**
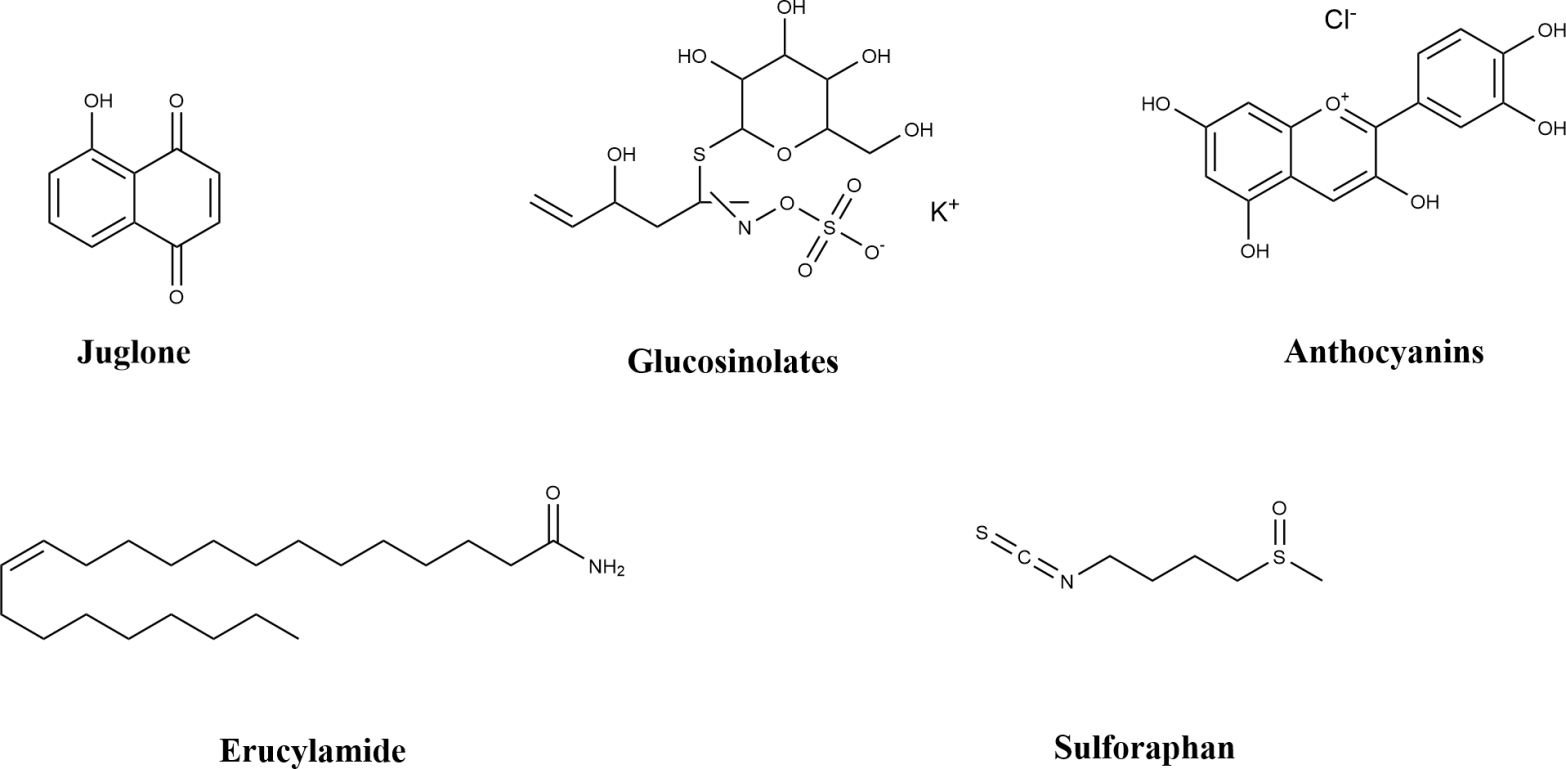
Chemical structure of other class compounds.

Sulforaphane (**Figure 6**) is an isothiocyanate generated by myrosinase-mediated hydrolysis of glucosinolates (Glu) in plants, with a chemical structure of 1-isothiocyanato-4R-(methylsulfinyl)butane. It exhibits diverse biological activities, including antioxidant, antibacterial, and anti-inflammatory properties, and exerts significant inhibitory effects on *P. syringae* (Wang et al., 2020). Its primary mechanism involves modifying the cysteine residue at position 209 of the HrpS protein in *P. syringae*, which effectively inhibits T3SS activity. As a complex secretion system facilitating the injection of effector proteins into host cells by bacterial pathogens, T3SS is essential for pathogenicity. Thus, this specific modification by sulforaphane substantially impairs the pathogenic capacity of *P. syringae.*

## 3 Control of *Pseudomonas syringae* by animal-derived natural products

### 3.1 Chitosan

**Figure 7:**
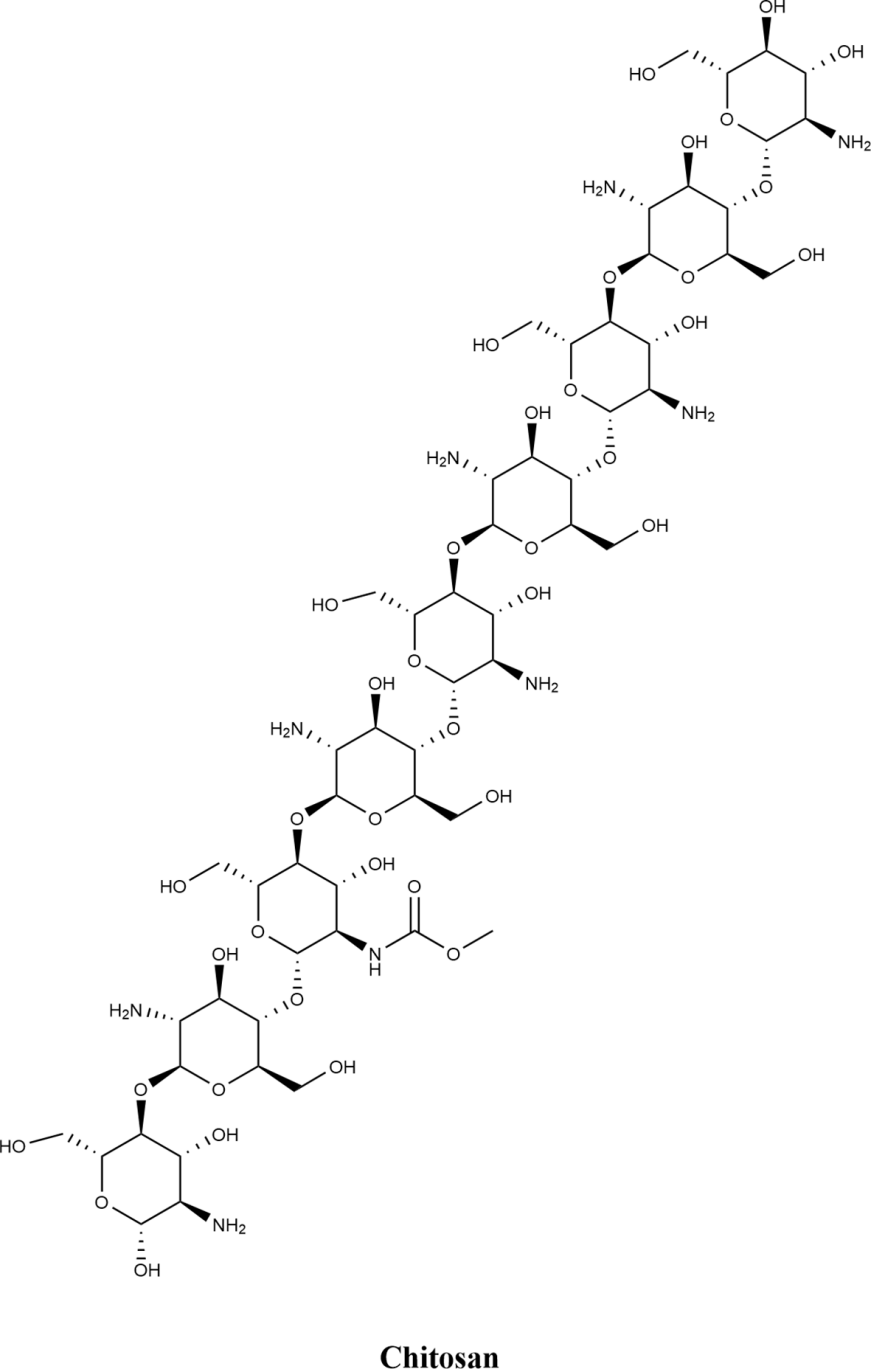
Chemical structure of natural products of animal origin.

Chitosan (**Figure 7**), also known as deacetylated chitin, is a natural linear polysaccharide composed of β-(1→4)-linked 2-amino-2-deoxy-D-glucose and small amounts of N-acetyl-D-glucosamine. Abundant hydroxyl and amino functional groups endow it with excellent biocompatibility and bioactivity. In controlling *P. syringae*, chitosan exerts antibacterial effects primarily by disrupting the bacterial cell wall and membrane: the amino and hydroxyl groups in its molecular structure interact with the negative charges on the bacterial cell wall, compromising cell wall integrity and further damaging the cell membrane. This disruption leads to leakage of intracellular contents, ultimately inhibiting bacterial growth and proliferation. For instance, Yan et al (Yan et al., 2024). found that a composite coating of chitosan (CTS) and curdlan (CUR) inhibited *P. syringae* proliferation in a concentration-dependent manner. Further scanning electron microscopy (SEM) observations revealed that the treated bacteria exhibited broken cell membranes, significant leakage of intracellular contents, and obvious morphological changes—these findings further confirm the destructive effect of the chitosan-based composite coating on the cellular structure of *P. syringae*.

Carboxymethyl chitosan (CMCS) is a carboxymethylated derivative of chitosan that exerts a dose-dependent inhibitory effect on the Gram-negative bacterium *P. syringae*. Its positively charged amino groups bind to the negatively charged phospholipids in bacterial cell membranes, disrupting membrane integrity and causing leakage of intracellular substances to achieve antibacterial activity (Matica et al., 2021). Additionally, CMCS can activate key enzymes in the plant phenylpropanoid metabolism (e.g., phenylalanine ammonia-lyase), promoting phytoalexin synthesis to enhance plant resistance (Li et al., 2022).Li et al (Li et al., 2022), for example, demonstrated that treatment with 0.5 mg/mL CMCS increased the H_2_O_2_ content in tomato leaves by 2.3-fold and the expression level of the PR1 gene by 4.7-fold, significantly reducing lesions induced by *P. syringae*.

### 3.2 Propolis

Propolis is a viscous solid colloid produced by bees (*Apis* spp.), formed by mixing plant resins with secretions from their maxillary and wax glands. Chemically, it is a complex mixture consisting mainly of resin, wax, flavonoids, phenolic acids, vitamins, and minerals—with flavonoids as one of its key active components. Propolis exhibits diverse pharmacological activities, including antibacterial, antiviral, antiparasitic, anti-inflammatory, antioxidant, and immunomodulatory effects. Studies have shown that flavonoids in propolis interact with bacterial cell wall components, compromising cell wall integrity and further damaging the structure and function of the cell membrane. This disruption leads to leakage of intracellular contents, ultimately inhibiting bacterial growth and proliferation. Ordóñez et al (Ordonez et al., 2011). compared six propolis samples from northern Argentina and evaluated the antibacterial activity of their extracts against *P. syringae*. They found that the T1-PE sample exhibited the highest activity, with 2, 4-dihydroxychalcone identified as its main antibacterial compound. Additionally, spraying a propolis solution on tomato fruits infected with *P. syringae* significantly reduced disease severity—further confirming the *in vivo* bactericidal effect of propolis.

### 3.3 Antimicrobial peptides

Antimicrobial peptides (AMPs) are naturally occurring small-molecule peptides with broad-spectrum antimicrobial activity. Typically consisting of 12–50 amino acids and having a molecular weight of 1–5 kDa, they are characterized by multi-targeting and multi-functional properties. Nuno et al (Mariz-Ponte et al., 2021a). investigated six AMPs (BP100, RW-BP100, CA-M, 3.1, D4E1, and Dhvar-5), among which BP100 and CA-M exhibited relatively strong inhibitory and bactericidal effects against *P. syringae*. Further flow cytometry analysis revealed that peptide 3.1 penetrated bacterial membranes more rapidly than the other tested AMPs. Tests on peptide mixtures also showed that the BP100:3.1 combination could effectively reduce *P. syringae* viability even at low concentrations. Additionally, Zhang Mingyu et al (Zhang et al., 2023). designed and synthesized a novel AMP (Jelleine-Ic) based on the natural peptide Jelleine-I, and this new peptide displayed enhanced antibacterial activity. Mechanistic studies indicated that it targets the *P. syringae* cell membrane: it increases membrane permeability and dissipates membrane potential, ultimately inducing intracellular calcium leakage in the bacteria.

## 4 Control of *Pseudomonas syringae* by microbial-derived natural products

### 4.1 Actinomycete-derived natural products

Wugufengsu (WGFs) are nucleoside-based small-molecule compounds isolated from the actinobacterium NEAU6. Studies have demonstrated that WGFs can effectively induce the early immune response in Arabidopsis thaliana and significantly enhance its resistance to *P. syringae*. Molecular mechanism analysis (Zhang and Xiang, 2021) reveals that WGFs activate the plant immune system through three pathways: first, they upregulate the expression of PAD4 (Phytoalexin Deficient 4) and SARD1 (Systemic Acquired Resistance Deficient 1)—key genes in the salicylic acid (SA) signaling pathway; second, they significantly increase the expression levels of genes associated with the synthesis of the phytoalexin camalexin, including *PAD3*, *CYP71A12*, and *CYP71A13*; third, they trigger the burst of reactive oxygen species (ROS) in plant cells.

### 4.2 Bacterial-derived natural products

*Methylobacterium* sp. is a symbiotic bacterium widely colonizing the plant phyllosphere, and its metabolites exhibit significant inhibitory effects on plant pathogens. The Oxford Cup method was used to assess the antibacterial activity of Methylobacterium sp. fermentation broth against *P. syringae*. Results showed that the inhibition zone diameter reached 21.30 ± 0.83 mm, confirming that secondary metabolites secreted by this bacterium possess potent inhibitory effects on the pathogen (Shi et al., 2023).

*Bacillus velezensis* ZF2 is a Gram-positive bacterium isolated and screened from cucumber plants. Studies have demonstrated that this strain exerts significant inhibitory activity against *P. syringae*, Botrytis cinerea (gray mold pathogen), and Fusarium oxysporum (fusarium wilt pathogen) (Zhao et al., 2019). Results from the plate confrontation assay and double-layer culture method confirmed that strain ZF2 has broad-spectrum antibacterial properties. Further detailed analysis of its antibacterial spectrum revealed an inhibition zone diameter of 5.2 ± 0.1 cm (inhibition rate: 58.10 ± 0.86%) against *P. syringae* pv. lachrymans (the pathovar causing angular leaf spot) and 4.9 ± 0.1 cm (inhibition rate: 54.03 ± 1.41%) against *P. syringae* pv. tomato (the pathovar causing tomato bacterial speck).

The extract of *Bacillus* sp. BR3 contains 2-phenylethylacetamide, L-phenyllactic acid, 11-methyl-11-hydroxydodecanamide, cyclo(leucine-leucine), and cyclo(L-valine-L-proline) dipeptides. This extract exerts specific inhibitory effects on the plant pathogen *P. syringae* and significantly impairs the function of the GacS/GacA two-component signaling system in *P. syringae* pv. tomato DC3000. Its mechanism involves inhibiting the transcription of the *gacS* gene (β-galactosidase activity assays showed reduced promoter activity) and the expression of the GacS protein (Western blot assays indicated decreased protein levels). This, in turn, affects the transcriptional levels of the downstream GacA-dependent small RNA genes *rsmZ* and *rsmY* (β-galactosidase activity was significantly downregulated in the wild-type strain but unchanged in the *gacA* mutant). Further transcriptomic analysis revealed that treatment with this extract also reduced the expression levels of Type III Secretion System (T3SS)-related genes *hrpL* and *avrPto* in *P. syringae*, confirming that it impairs the pathogen’s pathogenicity through multi-target intervention (Zhang et al., 2019).

### 4.3 Fungal-derived natural products

Saad et al (Saad et al., 2021). isolated 28 compounds from endophytic, soil, and marine fungi using spectroscopic techniques, and subsequently evaluated their anti-phytopathogenic and herbicidal activities against Echinochloa crus-galli (barnyard grass). Among these compounds, methyleurotinone exhibited broad-spectrum anti-phytopathogenic activity, with a particularly notable inhibitory effect on *P. syringae*—the minimum inhibitory concentration (MIC) against this bacterium was 125 mg/L.

(7S, 11S)-(+)-12-hydroxysydonic acid is a novel sesquiterpenoid isolated by Liu et al (Yang et al., 2023). from the ethyl acetate (EtOAc) crude extract of fermentation broth of the marine fungus *Aspergillus sydowii LW09*. The MIC of this compound against *P. syringae* was 32 μg/mL.

**Figure 8:**
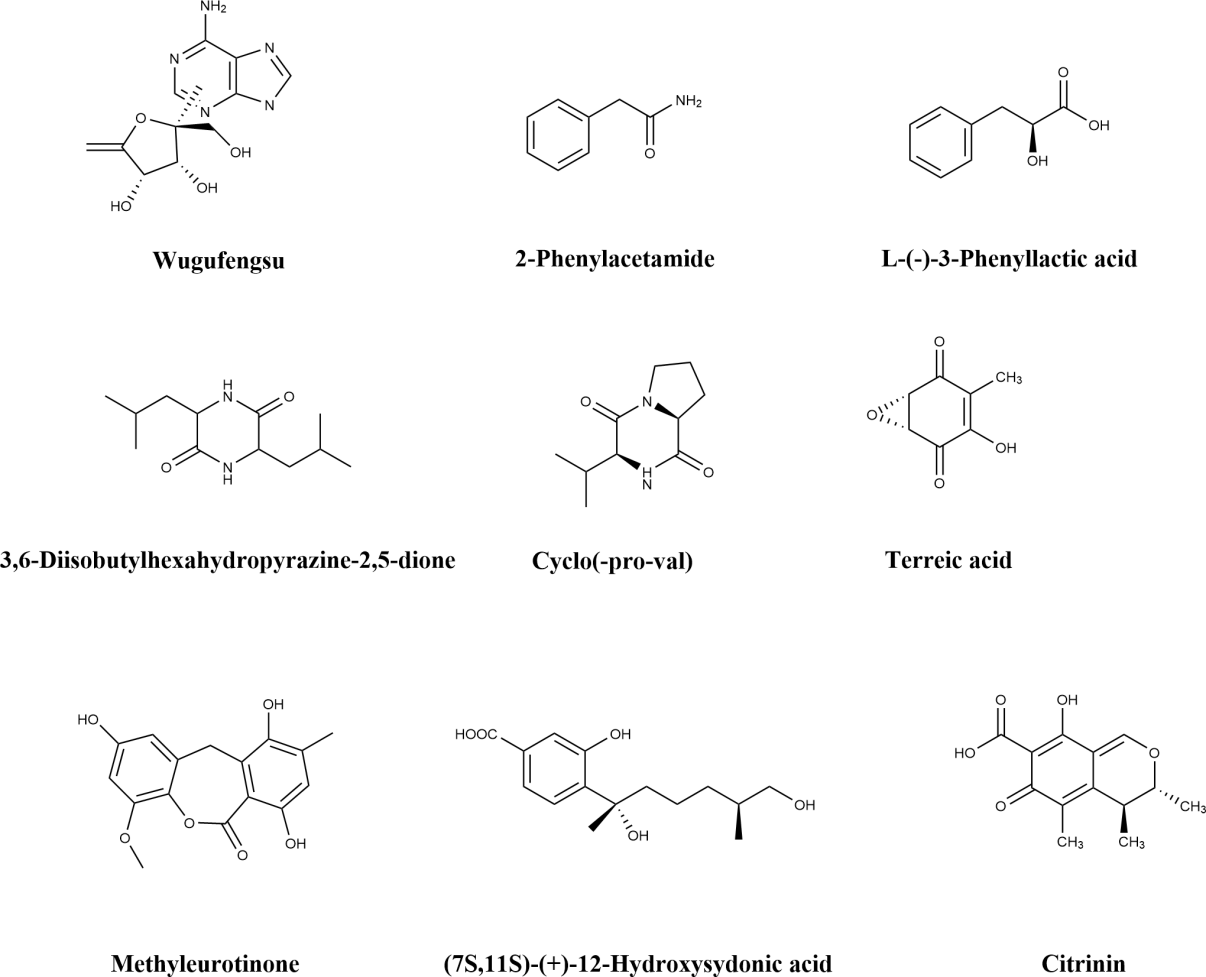
Chemical structure of natural products of microbial origin.

Zhang Yingluo et al (Kong et al., 2023). isolated 21 endophytic fungi from the roots, stems, leaves, and tubers of the medicinal plants *Pinellia ternata* and *Pinellia pedatisecta*; all isolated fungi showed antibacterial activity against pathogens including Escherichia coli, Staphylococcus aureus, and *P. syringae*. Additionally, seven compounds were purified from *Alternaria angustiovoidea* PT09 (an endophyte of *P. ternata*) and *Aspergillus floccosus* PP39 (an endophyte of *P. pedatisecta*).Among them, terreic acid and citrinin (**Figure 8**) showed potent inhibitory effects on *P. syringae*: terreic acid had an inhibition zone diameter (IZD) of 40.2 mm and a minimum inhibitory concentration (MIC) of 1.56 μg/mL, while citrinin had an IZD of 26.0 mm and an MIC of 6.25 μg/mL. These effects were either superior to or equivalent to those of the positive control, gentamicin sulfate.

Lin Libin et al (Lin et al., 2021). isolated 16 metabolites from the endophytic fungus *Aspergillus chevalieri* SQ-8, including 7 C7-alkylated salicylaldehyde derivatives and 9 prenylated indole alkaloids. Three of these metabolites were new compounds: aspergillin A, aspergillin B, and neoechinulin F. Specifically, aspergillin A and aspergillin B displayed antibacterial activity against *P. syringae* with an MIC of 6.25 μM. Further scanning electron microscopy (SEM) observations suggested that these two compounds may exert their antibacterial effects by altering the external structure of *P. syringae* and causing cell membrane rupture or deformation. Thus, aspergillin A and aspergillin B hold promise as potential candidates for agricultural fungicides.

## 5 Conclusion and prospect

### 5.1 Research summary

**Figure 9:**
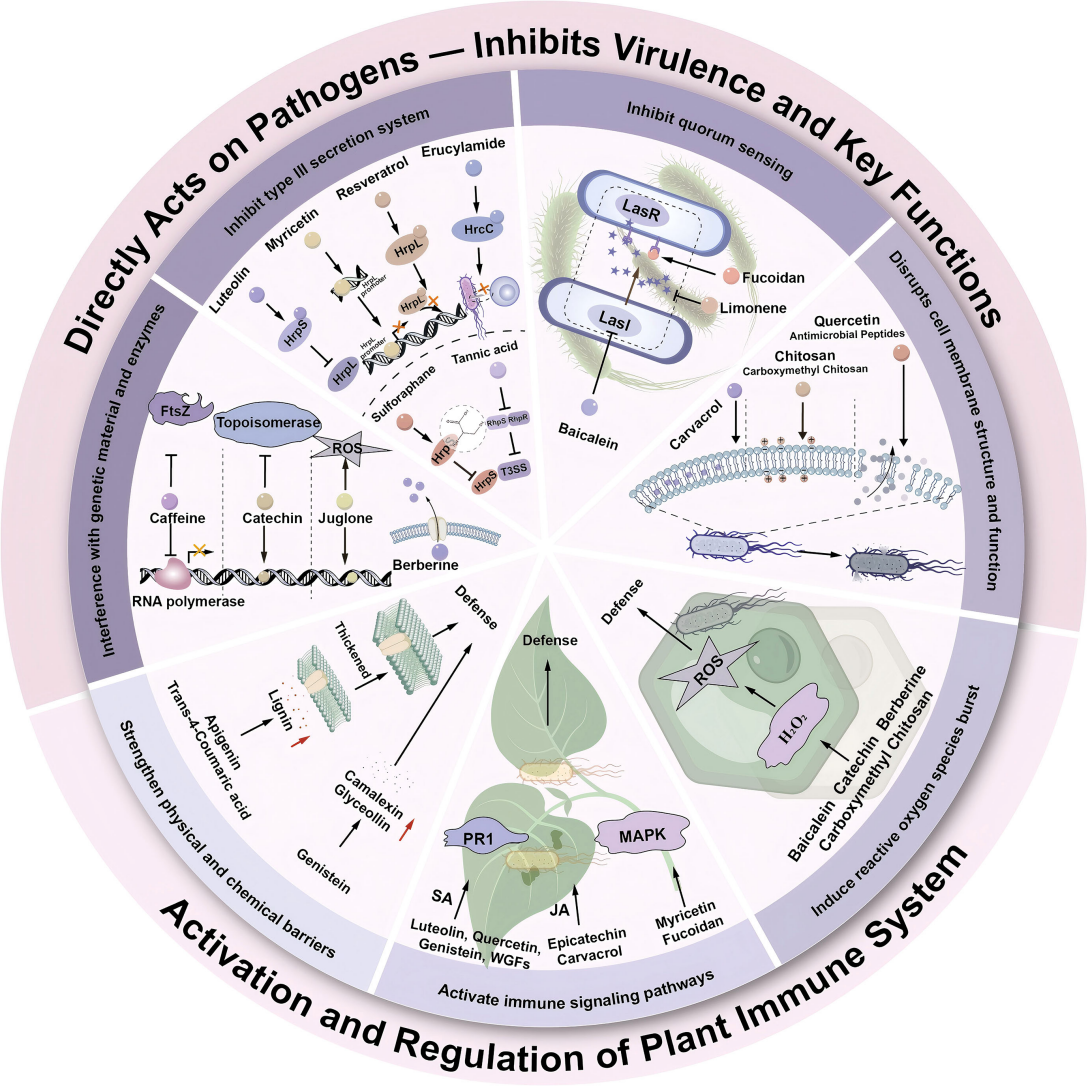
Plant-derived and microbial-derived natural products in controlling *Pseudomonas syringae*.

This article systematically reviews the research progress of plant-derived and microbial-derived natural products in controlling *P. syringae* (**Figure 9**). Plant-derived natural products exert antibacterial effects through multiple mechanisms, including inhibiting the pathogen’s Type III Secretion System (T3SS), disrupting cell membrane integrity, inducing reactive oxygen species (ROS) accumulation, and activating plant immune signaling pathways (e.g., salicylic acid (SA), jasmonic acid (JA), and systemic acquired resistance (SAR)). In contrast, animal-derived natural products typically act via more specific physicochemical modes of action. For instance, antimicrobial peptides (AMPs) primarily target microbial membranes through electrostatic interactions and pore formation, conferring broad-spectrum activity with a lower risk of resistance development (Zasloff, 2002; Wang et al., 2016). Chitosan and related polymers, on the other hand, function mainly through charge-mediated interactions and physical barrier formation; these compounds not only directly inhibit pathogens but also act as elicitors to enhance plant immunity (Kumar, 2019; Li et al., 2021).Microbial-derived natural products achieve synergistic pathogen control by regulating the host’s immune response and interfering with the pathogen’s quorum-sensing system. Additionally, several natural products can further suppress *P. syringae* by forming physical barriers, disrupting metabolic pathways, and modulating gene expression. Collectively, these studies demonstrate that animal-, plant-, and microbial-derived natural products possess distinct mechanistic characteristics, thereby providing a multi-layered theoretical basis and a rich candidate pool for developing green, sustainable strategies to manage plant diseases.

### 5.2 Future perspectives

The future of natural product pesticides as mainstream alternatives hinges on addressing key commercialization challenges—including technical hurdles such as poor stability and short persistence (Isman, 2020), higher production costs (Damalas and Koutroubas, 2018), and complex regulatory frameworks (Kumar et al., 2021)—while demonstrating their comprehensive cost-benefit advantages. A holistic assessment indicates that although direct application costs may be 20–50% higher, their value proposition is significantly enhanced by direct economic returns (e.g., product premiums), critical ecological benefits (e.g., reduced environmental impact and preserved biodiversity) (Pretty et al., 2018), and social gains (e.g., improved food safety). Studies show that integrating such sustainable practices can boost long-term farm profitability by 10–30% (Lechenet et al., 2017). Future progress will therefore depend on targeted research and development (R&D) to enhance product performance and affordability, coupled with policy reforms to establish streamlined, internationally harmonized regulations that facilitate market access and recognize their multifaceted benefits (Mosa et al., 2021).

While substantial progress has been made in researching natural products for *P. syringae* control, considerable room for improvement remains. With the continuous advancement of technologies such as genomics and proteomics, the interaction mechanisms between natural products and *P. syringae* can be analyzed more systematically and in depth—providing critical target support for the development of novel antibacterial agents. For example, a collaborative study by Professor Lei Xiaoguang’s team and Researcher Zhou Jianmin (Wang et al., 2020) demonstrated that erucylamide (a natural product from Arabidopsis thaliana) can specifically disrupt the assembly of *P. syringae*’s Type III Secretion System (T3SS), significantly inhibiting its pathogenicity and thereby achieving broad-spectrum control against multiple bacterial infections. Beyond known targets like T3SS, research can also explore how natural products regulate other key virulence factors or physiological processes of *P. syringae*—such as bacterial metabolic pathways, biofilm formation, and quorum sensing—to identify additional potential targets and expand ideas for developing novel antibacterial agents.

Following target identification, in silico approaches including molecular docking and molecular dynamics (MD) simulations can serve as powerful tools to accelerate the discovery process. These computational methods can predict the binding affinity and interaction modes of natural products or their derivatives with newly identified virulence targets (e.g., key enzymes in metabolic pathways, quorum-sensing regulators) (Spiegel et al., 2021; Niroula et al., 2025). This structure-based rational design strategy can prioritize the most promising candidates for subsequent *in vitro* and *in vivo* validation, thereby optimizing the efficiency of developing target-specific anti-*P. syringae* agents (Marcelletti and Scortichini, 2019).

Additionally, studies have noted that natural product extraction and isolation technologies face challenges such as low extraction efficiency, low purity, and poor stability (Qiu et al., 2022)—issues that hinder their practical application. Thus, further optimization of natural product extraction and processing technologies is needed to improve their stability and bioavailability. For instance, Zhang et al (Zhang et al., 2018). used enzyme-assisted extraction to isolate baicalein; optimizing the extraction process increased baicalein’s extraction rate and purity while significantly enhancing its stability. Furthermore, Liao et al (Liao et al., 2021). employed ultrasonic-assisted extraction to obtain flavonoids from peanut shells—this method not only improved extraction efficiency but also enhanced the products’ stability and bioavailability. In the future, natural product extraction processes can be further explored and optimized (e.g., through the use of novel extraction solvents or advanced extraction methods) to increase the efficiency and purity of natural product isolation. Additionally, chemical modification of natural products can help enhance their stability and bioactivity.

The development of multi-target antibacterial agents is a pivotal focus in contemporary antimicrobial research. Natural products often exhibit multi-target antibacterial properties, and the strategic combination of different natural compounds can yield synergistic effects that enhance antibacterial efficacy while effectively mitigating the emergence of pathogen resistance. In a study on treating kiwifruit bacterial canker caused by *P. syringae*, Nuno Mariz-Ponte et al. found that the combined use of multiple antimicrobial peptides produced significant synergistic antibacterial effects. The overall inhibitory activity was markedly higher than the sum of the activities of individual peptides, and it even showed synergistic improvements when combined with other treatments (Mariz-Ponte et al., 2021b). Acetosyringone (3, 5-dimethoxy-4-hydroxyacetophenone, AS), a syringyl-type phenolic compound, can depolarize bacterial cell membranes when mixed with hydrogen peroxide and peroxidase, thereby inhibiting the metabolism and proliferation of pathogenic mutant *P. syringae* and rapidly achieving bacteriostatic effects (Szatmári et al., 2021). Additionally, salicylic acid can reduce the drug sensitivity of *P. syringae*-induced diseases through a synergistic agonist-dependent mechanism, thereby inhibiting the development of its drug resistance (Shirasu et al., 1997).For example, a study by Hongkai Bi’s research team, published in Science Advances (Jia et al., 2023), elucidated a novel multi-target mechanism of action of the natural product chrysomycin A (ChryA) against methicillin-resistant Staphylococcus aureus (MRSA). This investigation not only highlighted the potential of natural products as multi-target antibacterial agents but also provided innovative conceptual frameworks and empirical evidence for the development of new antibacterial therapeutics. Future research should aim to further clarify the synergistic interactions between various natural products to identify optimal combinations that maximize synergistic effects, thereby facilitating the creation of highly effective multi-target antibacterial agents.

Finally, with strong national policy support for green agriculture and sustainable development, plus growing public attention to environmental protection and food safety, developing eco-friendly control measures has become an urgent priority. Thus, in the development and application of natural products for *P. syringae* control, emphasis should be placed on their ecological and environmental safety—this requires systematic studies on natural products’ environmental degradation pathways, residual levels, and impacts on non-target organisms to ensure environmental compatibility and sustainability during their use. Additionally, combining natural products with microbial fertilizers, plant nutrients, and other substances to form a comprehensive biological control system can further reduce chemical pesticide use and ease environmental burden. Through the aforementioned research and exploration, it is expected that more efficient, safe novel biological pesticides will be developed; this will promote the large-scale application of eco-friendly antibacterial agents in agricultural production and provide support for the green control of plant diseases and ecological environmental protection.
